# Successful Endovascular Therapy Using the Transtibial Approach in a Patient With a History of Iliofemoral and Femorofemoral Surgical Bypass

**DOI:** 10.7759/cureus.40837

**Published:** 2023-06-22

**Authors:** Daisuke Tokutake, Eiji Miyauchi, Ryo Arikawa, Naoya Oketani, Mitsuru Ohishi

**Affiliations:** 1 Department of Cardiology, Kagoshima City Hospital, Kagoshima, JPN; 2 Department of Cardiovascular Medicine and Hypertension, Kagoshima University Graduate School of Medical and Dental Sciences, Kagoshima, JPN

**Keywords:** access site, transankle intervention, transtibial intervention, endovascular therapy, posterior tibial artery, dorsalis pedis artery

## Abstract

Multiple stenotic lesions may restrict the access sites for endovascular therapy in the lower extremity arteries. Because guide sheaths used for endovascular therapy have recently become easier to insert, they are directly inserted into the posterior tibial or dorsalis pedis artery to perform the transtibial approach. We herein describe an 81-year-old man who was admitted to our hospital because of claudication of the left lower extremity. He had a history of left iliofemoral and femorofemoral bypass surgery. The patient’s symptom was due to a stenotic lesion extending from the left common femoral artery to the distal part of the left superficial femoral artery. In an angiographic procedure using the antegrade approach via the right radial artery, a multipurpose catheter became stuck in the middle of the left iliofemoral bypass. The antegrade ipsilateral approach was too close to the stenotic lesion for the insertion of the guide sheath. Therefore, a retrograde approach using a 5-French guide sheath inserted via the dorsalis pedis artery was successfully performed.

## Introduction

In endovascular therapy (EVT), the primary access site is the common femoral artery (CFA). However, Seto et al. [1] reported access site complications, such as retroperitoneal hemorrhage, pseudoaneurysm, and arteriovenous fistulas. In addition, Hashimoto et al. [2] reported that patients who undergo EVT have a high risk of bleeding. The transradial approach was shown to reduce the incidence of access site complications in patients who underwent percutaneous coronary interventions in a study by Ahsan et al. [3]. When performing EVT, however, this approach cannot be extended to the distal superficial femoral artery (SFA) or farther because of the device length. Sugihara et al. [4] reported that multivessel stenotic lesions limit the access site for EVT and that a history of surgical bypass to the lower limb may also limit the access site.

Liang et al. [5] described the use of tibial arteries as new access sites for EVT. We herein describe a case in which guide sheaths were directly inserted into the posterior tibial artery or dorsalis pedis artery to perform EVT using the transtibial approach.

## Case presentation

An 81-year-old man was referred to our hospital because of intermittent claudication of the left lower limb that had gradually worsened during the previous four months. He was a previous smoker (20 cigarettes/day for 40 years) and had stopped smoking 20 years before presentation. He had a history of EVT with stenting in the middle of the left SFA and two-stage prosthetic bypass grafting for the left common iliac artery-CFA and left CFA-right CFA bypass at another hospital about 10 years previously.

On physical examination, his height was 164 cm, weight was 66.4 kg, and body mass index was 24.5 kg/m^2^. There were no wounds or cyanosis in his left lower limb. The popliteal artery was faintly palpable, but the posterior tibial and dorsalis pedis arteries were not palpable. Surgical scars were observed in both groin regions. His ankle-brachial index was 0.38 on the left side and 0.80 on the right side.

The patient had no history of diabetes, dyslipidemia, coronary artery disease, or cerebrovascular disease. His hemoglobin A1C (HbA1C) concentration was 5.9%, low-density lipoprotein cholesterol concentration was 121 mg/dL, high-density lipoprotein cholesterol concentration was 48 mg/dL, and triglyceride concentration was 94 mg/dL without medications. He had chronic kidney disease; his creatinine concentration was 1.18 mg/dL and estimated glomerular filtration rate was 45.9 mL/min/1.73 m^2^.

**Figure 1:**
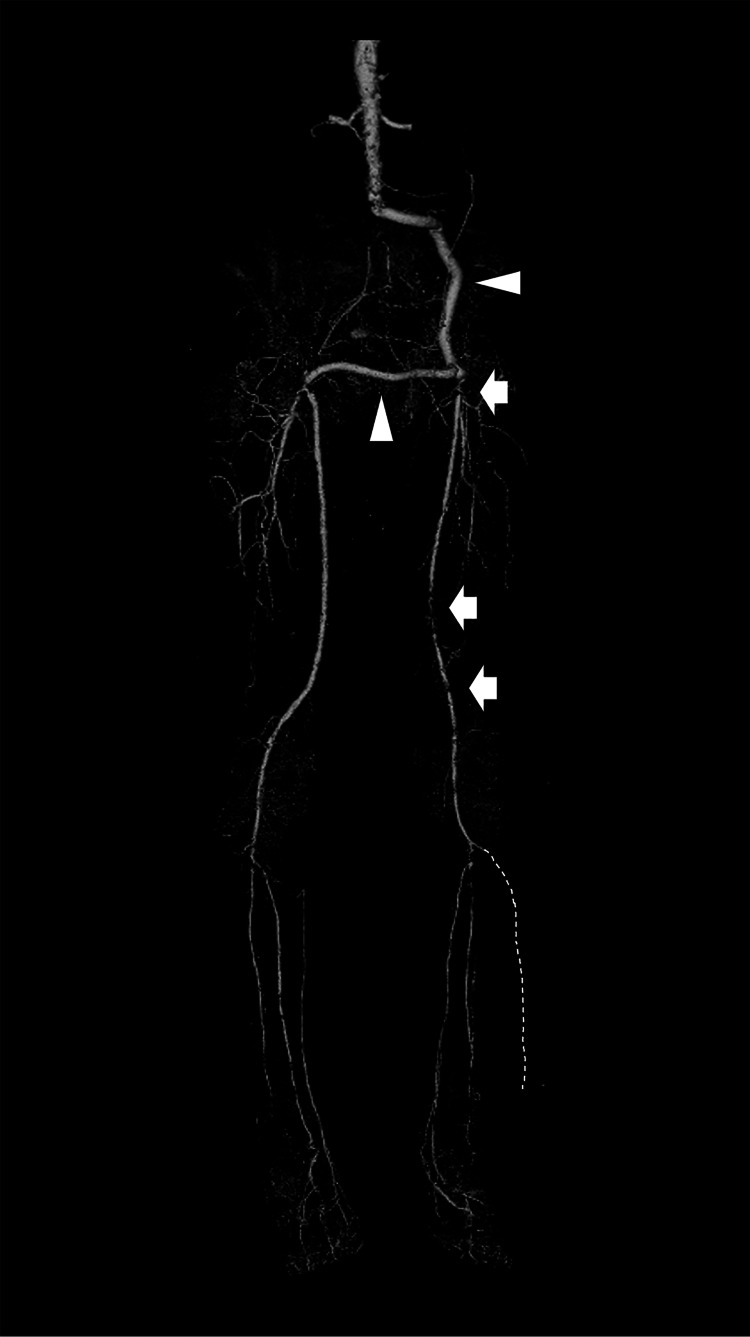
Three-dimensional constructed computed tomography angiography The white arrowheads indicate the prosthesis bypass grafts (femorofemoral and left iliofemoral) without stenotic lesions. The white arrows indicate the stenotic lesions in the left common femoral artery and in the middle and distal parts of the left superficial femoral artery. The left anterior tibial artery was completely occluded in the proximal part as indicated by the white dotted line.

Contrast-enhanced computed tomography revealed no stenotic lesions in either of the two prosthesis bypass grafts; however, stenotic lesions with calcification were detected from the left CFA to the distal part of the SFA (Figure 1).

In addition, the proximal part of the left anterior tibial artery (ATA) was completely occluded. The patient was diagnosed with left lower extremity artery disease, and his symptom was Fontaine classification stage IIb and and Rutherford classification category 3. Then, we determined that revascularization for his left limb was recommended. After discussing the patient’s condition with our vascular surgeons, we considered that performing a re-bypass was not preferable because of adhesions at the inguinal anastomosis. Therefore, EVT was performed.

Management and outcome

**Figure 2:**
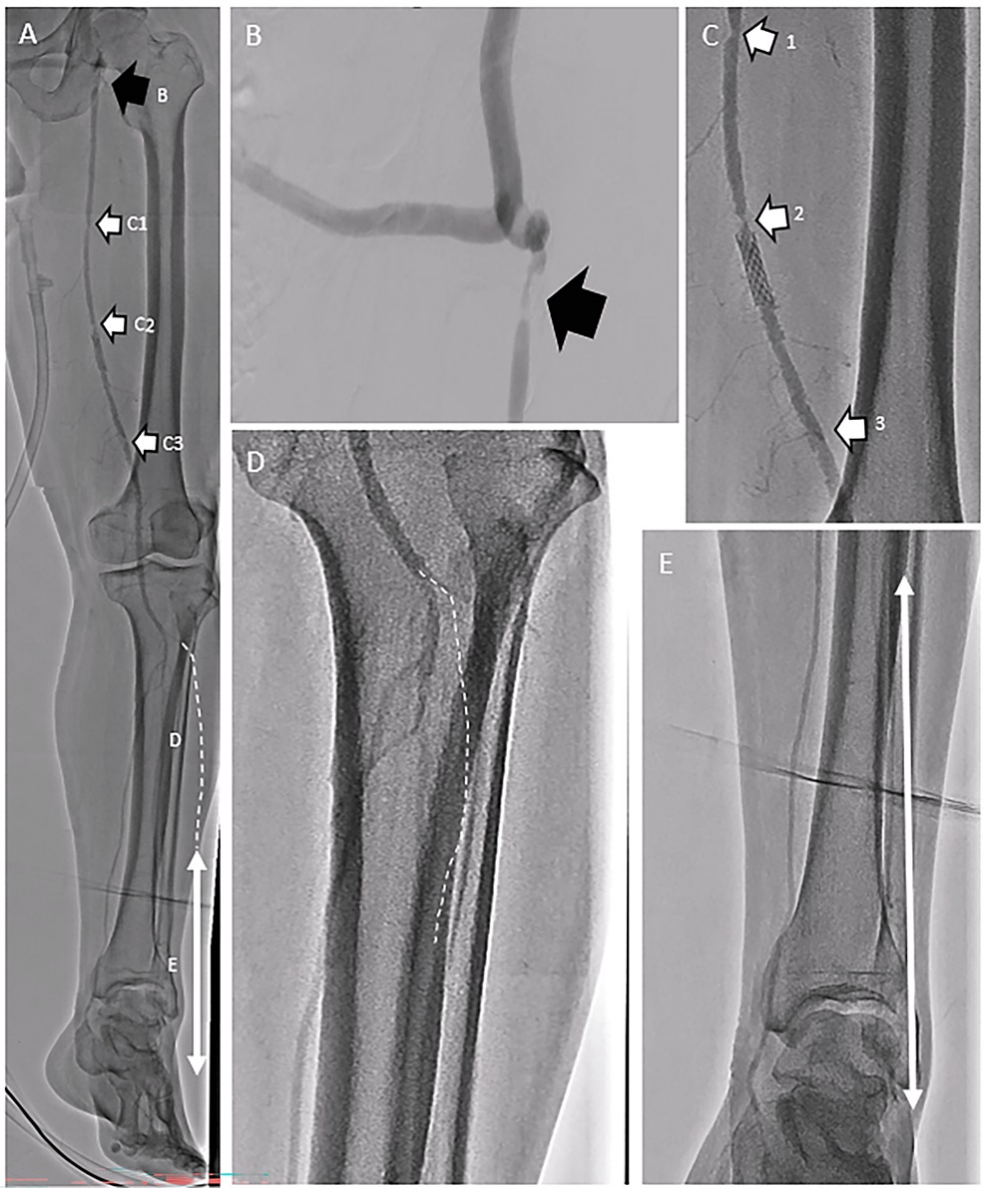
Angiography prior to endovascular therapy (A) Overview of the left lower extremity arteries. (B) Stenotic lesion extending from the left common femoral artery to the ostium of the superficial femoral artery. As indicated by the black arrow, a severely stenotic lesion was observed in the left common femoral artery immediately distal to the anastomosis of the prosthesis bypass graft. (C) Stenotic lesions of the superficial femoral artery. As indicated by the white arrows, severely stenotic lesions from the middle to distal parts of the superficial femoral artery were detected. (D) Early phase of contrast enhancement of below-the-knee arteries. As indicated by the white dotted line, total occlusion at the proximal part of the anterior tibial artery was observed. (E) Delayed phase of contrast enhancement of below-the-knee arteries. The distal part of the left anterior tibial artery and left dorsalis pedis artery showed contrast via the collateral vessels from the perforator branches of the left peroneal artery.

To determine the optimal EVT strategy, we performed angiography from the entrance of the left iliofemoral prosthesis bypass graft using a multipurpose catheter inserted via the right radial artery. Multiple stenotic lesions were observed from the left CFA to the distal part of the left SFA (Figure 2A-C).

The multipurpose catheter was stuck in the middle of the left iliofemoral prosthesis bypass graft, and we considered that an antegrade approach via the right radial artery would be impossible. In addition, an ipsilateral approach via the left iliofemoral prosthesis bypass graft was too close to the stenotic lesion for insertion of the guide sheath. Moreover, puncturing the prosthesis bypass graft might have increased the risk of bleeding and/or infectious complications [4]. Therefore, we chose the retrograde approach; an approach via the left popliteal artery was too close to the stenotic lesion. Prior angiography revealed that the flow in the left posterior tibial artery was maintained, the proximal part of the left ATA was occluded, and the distal part of the ATA and left dorsalis pedis artery exhibited contrast via the collateral vessels (Figure 2D, E). We then used a retrograde approach via the left dorsalis pedis artery and ATA to avoid reducing the blood flow via the left posterior tibial artery. The luminal diameter was 2.8 mm for both the left dorsalis pedis and distal part of the posterior tibial artery as estimated with a duplex ultrasound, and insertion of guide sheaths was thus considered possible.

**Figure 3:**
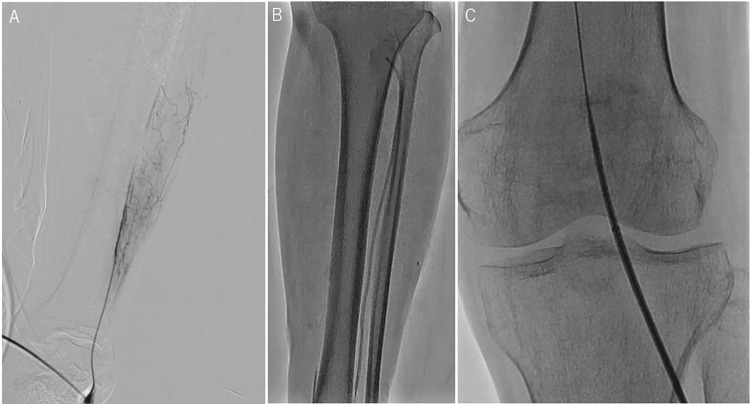
Steps of guide sheath insertion through the left dorsalis pedis artery (A) First, the left dorsalis pedis artery was punctured under duplex ultrasound guidance, and a 3-French sheath was inserted. (B) Second, a 0.014-inch guide wire was passed through the occluded lesion of the anterior tibial artery, which was reopened with standard balloon angioplasty using a 2.5- × 220-mm balloon. (C) Third, the 3-French sheath was replaced with a 5-French 53-cm sheath, and the guide sheath tip was placed at the middle part of the popliteal artery.

The left dorsalis pedis artery was punctured under duplex ultrasound guidance, and a 3-French 76-mm sheath (NAVIGATOR; Medikit, Tokyo, Japan) was inserted (Figure 3A).

A 0.014-inch wire (Gladius MG; Asahi Intecc, Aichi, Japan) was inserted to pass through the occlusive lesion of the ATA with the support of a 0.014-inch compatible microcatheter (Prominent Advance NEO 2; Tokai Medical Products, Aichi, Japan). The occlusion of the ATA was reperfused by standard balloon angioplasty using a 2.5- × 220-mm balloon (Coyote; Boston Scientific, Marlborough, MA, USA) (Figure 3B). We replaced the 3-French sheath with a 5-French 53-cm guide sheath (Parent Select 5082; Medikit), and the tip of the guide sheath was placed at the middle part of the popliteal artery (Figure 3C).

**Figure 4:**
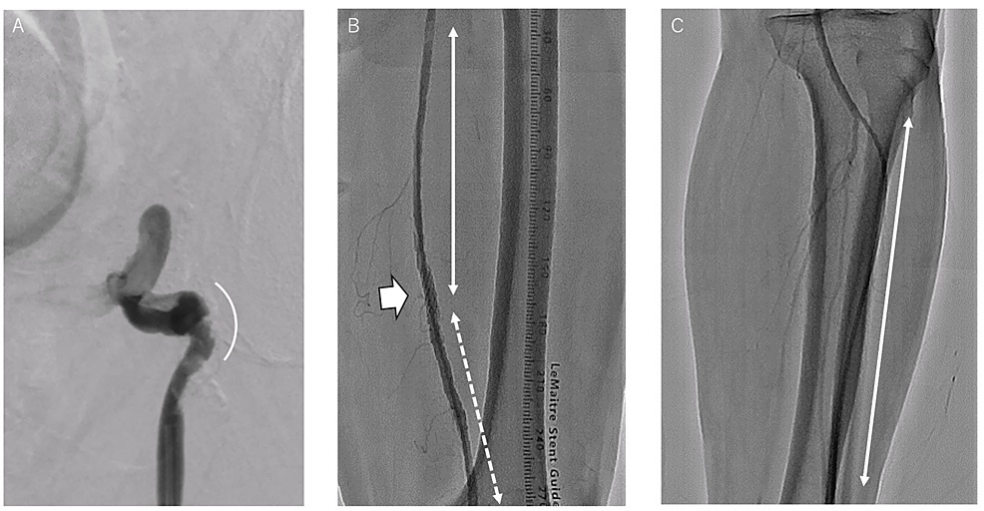
Angiography after endovascular therapy (A) As indicated by the white bracket, a severely stenotic lesion was dilated by standard balloon angioplasty, and a drug-coated balloon was placed to prevent restenosis. (B) As indicated by the white arrow, a short stent had been implanted at another hospital. As indicated by the white line, the stenotic lesion of the proximal part of the left superficial femoral artery was dilated with a drug-coated balloon after standard balloon angioplasty. Additionally, as indicated by the white dotted line, a bare metal stent was implanted after standard balloon angioplasty in the distal part of the left superficial femoral artery. (C) As indicated by the white line, the anterior tibial artery was totally contrasted.

After consecutive standard balloon angioplasty procedures, a drug-coated balloon (Ranger; Boston Scientific, USA) was placed from the left CFA to the middle part of the left SFA, and an interwoven stent (Supera; Abbott Laboratories, Chicago, IL, USA) was placed at the distal part of the left SFA (Figure 4).

The EVT procedure was completed with no complications. The claudication disappeared immediately. The ankle-brachial index on the left side was maintained at 0.96, and no restenotic lesions were observed using duplex ultrasound.

## Discussion

We have herein reported a case of EVT in a patient with a history of surgical bypass using a retrograde approach via the dorsalis pedis artery and ATA. In this case, choosing the access site that was the most considerable, the optimal transtibial approach was possible because of the improved guide sheath.

The reported diameters of the dorsalis pedis artery and posterior tibial artery close to the ankle joint are 2.8 and 2.4 mm, respectively [6]. The outer diameters of most delivery systems of stents and balloons for EVT are usually less than 2.1 mm. Therefore, the guide sheaths used in the transtibial approach must have an outer diameter of less than 2.4 mm and inner diameter of more than 2.1 mm. The total thickness of the sheath on both sides must therefore be less than 0.3 mm (less than 0.15 mm on each side). Although a sheath with a length of 16 cm was used in a previous report of the transtibial approach [7], the tip of the sheath could not reach the femoropopliteal lesions. In the transradial approach, protection using a long hydrophilic-coated sheath can help prevent radial spasm by significant catheter friction during manipulation [8]. Furthermore, removal of the coated sheath requires less force than an identical uncoated sheath [9]. Therefore, even with the transtibial approach, the pathway from the access site to the lesion should be protected by a hydrophilic-coated guide sheath as long as possible to reduce catheter friction and facilitate easy removal. Guide sheaths have recently been improved for direct insertion into the tibial artery, allowing the transtibial approach to be performed more safely and easily.

The transtibial approach for infrainguinal revascularization appears to be a reasonable approach with a high success rate and low complication rate and with a significant reduction in the radiation and contrast doses [10]. Furthermore, radial hemostasis devices can effectively achieve hemostasis following the transtibial approach with low rates of complications [11]. These findings should be further studied in randomized controlled trials, but the transtibial approach should be considered when the other sites are unsuitable.

## Conclusions

We successfully performed EVT using a retrograde transtibial approach for a patient in whom the antegrade approach was not possible because of a history of bypass surgery in the lower limb. The transtibial approach has become available owing to the improvement of the devices. Hence, we should consider the access sites in each patient for safety.
